# Vasectomy reversal and prostate cancer risk: A multi-centre collaborative demonstration project of the Intentional Population Data Linkage Network: Letter to the International Journal of Population Data Science

**DOI:** 10.23889/ijpds.v3i1.730

**Published:** 2018-10-08

**Authors:** James Boyd, Sean Randall, Emma Fuller

**Affiliations:** 1 Centre for Data Linkage, School of Public Health, Curtin University

## Abstract

This first collaborative demonstration project of the International Population Data Linkage Network (IPDLN) has recently been completed. This project collated data from five data linkage centres across Australia, the United Kingdom and Canada to investigate the effect of vasectomy reversal on prostate cancer risk in vasectomized men. We discuss the study and the challenges of organising and analysing multi-centre linked data studies.

In December 2008, the inaugural meeting of the International Health Data Linkage Network (now the International Population Data Linkage Network or IPDLN), was held in London and attended by over 30 participants from data linkage centres around the globe. Discussion at the meeting included the instigation of a collaborative demonstration project, in which data from multiple linkage centres would be combined to answer research questions not possible with data from a single centre alone.

At the meeting, members chose to replicate a Western Australian linkage study performed in 2004 whose results were deemed inconclusive due to a lack of power (1). This study investigated a link between prostate cancer and vasectomy by examining the effect of vasectomy reversal on prostate cancer risk in vasectomized men.

The issue of detection bias has been a key criticism of studies investigating prostate cancer risk in men with and without vasectomy, as it is likely that men who have undergone vasectomy will have greater screening for prostate cancer, leading to higher rates of diagnosis. The issues around detection bias have been addressed using a study design which compares the risk of prostate cancer in men with vasectomies and vasectomy reversals i.e. if vasectomy increases the risk of prostate cancer, then those with a subsequent vasectomy reversal should have a reduced risk of prostate cancer (1).

We are pleased to announce that the results of this project have now been published in The Journal of Urology (2). The study collated data from five data linkage centres (3); the Western Australian Data Linkage Branch; the Centre for Health Record Linkage in New South Wales, Australia; the Institute for Clinical Evaluative Sciences in Ontario, Canada; the Information Services Division, Scotland, United Kingdom and the SAIL Databank, Wales, United Kingdom. Application for data, ethics, and data analysis was completed locally by researchers using a shared analysis plan, with aggregate results collated in a meta-analysis. In total, there were 684,660 men with a vasectomy, of which 9,754 had a reversal. Combined analysis showed no protective effect of vasectomy reversal on risk of prostate cancer (HR, 95%CI: 0.92, 0.70-1.21), supporting previous studies which had found little or no evidence of a link between vasectomy and prostate cancer.

A key challenge in using multi-centre linked data lay in understanding the differences and similarities between health systems and data collections. In the present study a key issue was the coverage of vasectomy and vasectomy reversal procedures in each region’s data collection; vasectomy could be performed in primary care or in hospitals; vasectomy reversals could be performed privately or through the public system. Regions differed in whether private procedures were recorded in administrative datasets, whether certain procedures were available through the public system, whether primary care procedures were recorded, or whether a significant private healthcare system existed at all.

The lengthy period from study instigation to completion (nearly ten years) is largely not a result of the complexity of organising or conducting a multi-centre linked data study, but was due to more mundane considerations. This study was carried out without any funding, with work carried out on top of existing workloads, and with any costs absorbed by the centres. This meant that time available for the study was limited. In addition, key staff moved to new roles partway through the project with no available staff to continue. Due to logistical and funding issues, a number of other regions considered but could not partake in the final study, including England (Oxford Record Linkage Study), Manitoba (Manitoba Centre for Health Policy), Calgary (University of Calgary), and British Columbia (Centre for Health Services and Policy Research).

While challenges exist, this project demonstrated the utility and feasibility of multi-centre collaborative studies utilizing linked administrative data fostered through the IPDLN. Linked data studies have enormous benefits for population health research and significant implications for instigating change in current health policy and inciting the development of new policy. The extension of these methods to multi-centre multi-country studies further extends the range of research questions which can be answered. Studies which investigate rare conditions and diseases, studies with expected small effect sizes and studies focusing on international comparisons can all be best addressed using these methods. We encourage researchers who may benefit from utilizing multi-centre linked data studies to do so.

## Ethics

The authors declare that they have no conflicts of interest.

## References

[1] Borgmeier I, Holman CaJ. Does vasectomy reversal protect against prostate cancer? Annals of epidemiology. 2004;14(10):748-9, 10.1016/j.annepidem.2003.12.003 15519896

[2] Randall S, Boyd J, Fuller E, Brooks C, Morris C, Earle CC, et al. The Effect of Vasectomy Reversal on Prostate Cancer Risk: International Meta-Analysis of 684,660 Vasectomized Men. The Journal of urology. 2018;200(1):121-5, 10.1016/j.juro.2018.03.005 29524505

[3] Boyd JH, Randall SM, Ferrante AM. Application of privacy-preserving techniques in operational record linkage centres. Medical Data Privacy Handbook: Springer International Publishing; 2015. p. 267-87, 10.1007/978-3-319-23633-9_11

